# Impact of dizziness on migraine interictal burden in patients with vestibular migraine

**DOI:** 10.3389/fneur.2025.1723725

**Published:** 2025-12-12

**Authors:** Katsuhiro Adachi, Naoto Sakai, Kazuhiro Kimpara, Daiki Takahashi, Shinichi Arizono

**Affiliations:** 1 Sakai Neurosurgical Clinic, Hamamatsu, Japan; 2 School of Rehabilitation Science, Seirei Christopher University, Hamamatsu, Japan

**Keywords:** vestibular migraine, migraine interictal burden, MIBS-4, Dizziness Handicap Inventory, Video Head Impulse Test

## Abstract

**Introduction:**

Vestibular migraine (VM) remains an under-recognized condition despite its relative high prevalence and substantial impact on the interictal burden of migraine. We quantified vestibular dysfunctions and interictal migraine burden and evaluated whether dizziness contributes to the migraine interictal burden of VM.

**Methods:**

We conducted a retrospective observational study of consecutive patients fulfilling the Bárány Society and the *International Classification of Headache Disorders, 3rd edition*, criteria for VM who attended our outpatient clinic between December 2024 and April 2025. Multidimensional assessments included the Headache Impact Test-6 (HIT-6), Migraine Interictal Burden Scale-4 (MIBS-4), Dizziness Handicap Inventory (DHI), Self-Rating Depression Scale (SDS), Video Head Impulse Test (v-HIT), and posturography (Romberg ratio). Association between MIBS-4 and the other assessments were analyzed using Spearman’s correlation and stepwise multiple linear regression.

**Results:**

Seventy-five patients were included {median age 37 years [interquartile range (IQR): 27–47]; 74.3% female; 26.7% with aura}. The median HIT-6 score was 62 (IQR: 58–65) and the median MIBS-4 was 4.0 (IQR: 1.0–7.0), with 41.3% of patients exhibiting severe interictal burden (MIBS-4 ≥5). The median DHI score was 20 (IQR: 12–36), and the Romberg ratio was 1.45 (IQR: 1.03–1.97). Vestibular-ocular reflex gains were largely normal, whereas 73.3% of patients exhibited catch-up saccade (CUS) abnormalities. In univariable analyses, MIBS-4 correlated positively with HIT-6 (*ρ* = 0.414, *p* < 0.001), DHI (*ρ* = 0.419, *p* < 0.001), and SDS (*ρ* = 0.433, *p* < 0.001). In multivariable analysis, high HIT-6 scores (*β* = 0.265, *p* = 0.016) and high DHI total scores (*β* = 0.250, *p* = 0.019) independently predicted high MIBS-4.

**Conclusion:**

In patients with VM during the interictal period, vestibular functions were largely normal except for abnormalities in the v-HIT CUS, whereas subjective dizziness assessed by DHI significantly contributed to the high degree of migraine interictal burden. The dizziness experienced in VM resembled that of persistent postural-perceptual dizziness. These results indicate that vestibular rehabilitation might be effective in alleviating migraine interictal burden in patients with VM.

## Introduction

Migraine is a highly prevalent neurological disorder affecting approximately one billion people worldwide, with a higher prevalence in females (1, 2). It imposes a considerable burden on individuals and society, ranking as the third highest condition with the age-standardized disability-adjusted life-years according to t*he Global Burden of Disease Study 2021* (2). Recently, increasing attention has focused on migraine interictal burden, including functional limitations, activity avoidance, and anticipatory anxiety between migraine attacks. To quantify this aspect, the migraine interictal burden scale-4 (MIBS-4) was developed by Lipton et al. (3).

Vestibular migraine (VM), characterized by the coexistence of dizziness and balance disturbance with migraine, is among the most common causes of recurrent vertigo (4). Despite its prevalence and high impact on healthcare cost and utilization, VM remains an under-recognized condition with largely unknown pathophysiology (5). Although VM is characterized by recurrent attacks of dizziness, vestibular function tests during the interictal period often reveal no apparent abnormalities (6–8). Nevertheless, many VM patients report persistent subclinical symptoms such as mild dizziness, imbalance, and visual motion hypersensitivity, even outside of acute attacks (9). These findings suggest that VM may not be purely paroxysmal, and that substantial interictal burden related to dizziness may exist, potentially affecting quality of life despite normal vestibular test results.

Evaluating migraine interictal burden using the MIBS-4 may thus provide a more comprehensive understanding of VM and guide interventions aimed at alleviating persistent symptoms between migraine attacks. In this study, we assessed dizziness and vestibular dysfunctions during the interictal period of VM through multidimensional evaluations, including the Headache Impact Test-6 (HIT-6), MIBS-4, Dizziness Handicap Inventory (DHI), Self-Rating Depression Scale (SDS), Video Head Impulse Test (v-HIT), and posturography (Romberg ratio). We quantified vestibular dysfunction and interictal migraine burden and examined whether dizziness contributes to migraine interictal burden in VM using Spearman correlation and stepwise multiple linear regression.

The primary objective of this study was to determine whether subjective dizziness assessed by the DHI contributes to the MIBS-4 in patients with VM during the interictal period.

## Methods

### Study design and ethics

This retrospective observational study was conducted using the electronic medical records of Sakai Neurosurgical Clinic. Approval was obtained from the Sakai Neurosurgical Clinical Research Ethics Committee (Approval Number: SNC2025-01). Consecutive patients who visited the clinic between December 2024 and April 2025 and met the diagnostic criteria for VM proposed by the Bárány Society and the *International Classification of Headache Disorders, 3rd edition* (ICHD-3) (10) were included. These criteria require at least five episodes of vestibular symptoms temporally associated with migraine features, with other causes excluded, such as Meniere’s disease, benign paroxysmal positional vertigo, vestibular neuritis, vestibular schwannoma, cerebrovascular disease, infectious disease, demyelinating disease, degenerative disorders, or psychiatric disorders. To rule out intracranial pathology, all participants underwent magnetic resonance (MR) imaging and MR angiography to confirm the absence of intracranial lesions. Comprehensive medical histories were collected for each participant, including age, sex, migraine type (with or without aura), interval (months) from migraine onset to dizziness onset, dizziness symptoms (vertigo, unsteadiness or both), and the number of headache days, unclear days, and crystal-clear days per month. Crystal-clear days were defined as days without headache and with minimal or no migraine symptoms, whereas unclear days were defined as headache-free days that were not crystal-clear (11).

No formal *a priori* sample size calculation was performed: instead, all consecutive patients diagnosed with VM during the study period were included in the study.

### Migraine symptoms scales

The HIT-6 was used to assess the severity and impact of migraine and other severe headaches (12). It consists of six questions evaluating the frequency in the past 4 weeks of: (1) severe headache pain, (2) limitations in daily activities, such as household tasks, work, school, or social engagement, (3) the desire to lie down during a headache, (4) fatigue affecting work or daily activities, (5) irritability or frustration due to headache, and (6) difficulty concentrating on work or daily activities. Each item offers five responses options, namely,” never,” “rarely,” “sometimes,” “very often” and “always,” scored as 6, 8, 10, 11, and 13 points, respectively. The total score ranges from 36 to 78, with higher scores indicating a greater impact of headaches on daily functions. Interpretation of the total score is as follows: ≤49: minimal to no impact; 50–55: some impact; 56–59: substantial impact; 60–78: sever impact. The HIT-6 has demonstrated high internal consistency and reliability among patients with migraine (13–15).

The MIBS-4 was used to assess interictal migraine-related burden across four domains: (1) work or school functioning, when not experiencing headache, (2) worry about planning social or leisure activities, (3) overall life impact between attacks, and (4) feeling of helplessness in the absence of headache. Each item is rated on a six-point scale: “Do not know/NA” (0), “Never” (0), “Rarely” (1), “Some of the time” (2), “Much of the time” (3), and “Most of all of the time” (3). Total scores range from 0 to 12, with burden severity classified as none (0), mild (1–2), moderate (3–4), and severe (5–12).

### Neurotological examinations in interictal period of migraine

Neurotological examinations were performed during the interictal period of migraine, consisting of eight tests: Dizziness Handicap Inventory (DHI), Video Head Impulse Test (v-HIT), posturography, Dix–Hallpike test, Roll test, single-leg stance time, and 3-m Timed Up and Go (TUG) test. The DHI is a 25-item self-assessment scale designed to evaluate the self-perceived handicap caused by dizziness. Responses are scored as 4 for “yes,” 2 for “sometimes,” and 0 for “no.” The total DHI score ranges from 0 (no perceived handicap) to 100 (significant perceived handicap) (16), and can be categorized as no (0–14), mild (16–26), moderate (28–44), and severe (46–100) perceived handicaps (17). We also examined the Self-Rating Depression Scale (SDS), which was developed by Zung (18) and consists of 20 self-reported items derived from factor-analytic studies of depression. Total raw scores range from 20 to 80, with higher scores indicating greater depression symptom severity. An index score ≥50 is considered the cutoff for identifying adults with depression disorder.

The v-HIT is a physiological assessment tool used to evaluate the vestibulo-ocular reflex (VOR) at high frequency for each semicircular canal by calculating the duration ratio between the head impulse and the gaze deviation (19). A v-HIT-measured VOR gain <0.7 for a given semicircular canal has been reported as indicative of vestibular hypofunction (20). In this study, v-HIT assessments were performed using a high-speed binocular eye tracker (Interacoustics; EyeSeeCam). Vestibular dysfunction was defined as a VOR impairment <0.8 for the lateral semicircular canal, <0.7 for the anterior and posterior canals. Catch-up saccades (CUS), including covert, anti-covert, and overt saccades, were evaluated in the lateral semicircular canals with normal and reduced VOR gain. Posturography is used to measure changes in the center of pressure, representing the point of the entire pressure exerted by the foot-ground contact surface on the force plate. Graphical representations, known as stabilograms and statokinesigrams are obtained by measuring body sway using posturography (21). Body sway primarily reflects abnormalities in the utricle, an otolithic organ responsible for detecting horizontal linear acceleration and maintaining postural stability. We used a gravicorder (Anima; GW-31) to measure posturography and recorded the sway-path area, mean velocity of center-of- pressure movement over 60 s, total sway path length under open- and closed-eye conditions, and the Romberg ratio (eyes closed/eyes open). The Dix–Hallpike and Roll tests are diagnostic maneuvers used to determine whether otoconia have migrated into the semicircular canals, causing benign paroxysmal positional vertigo.

### Statistical analysis

All statistical analyses were conducted using IBM SPSS Statistics for Windows, version 24.0 (IBM Corp., Armonk, NY, United States). Descriptive statistics were calculated for all variables. Spearman’s rank correlation coefficients were used to evaluate the associations between MIBS-4 scores and clinical and neurotological variables. To identify independent predictors of MIBS-4 scores, we performed stepwise multiple linear regression analysis with MIBS-4 as the dependent variable. All clinical and neurotological variables listed in Table 1 were considered as candidate predictors, and variables were selected using a stepwise procedure (probability of *F* to enter ≤0.05 and to remove ≥0.10). A two-tailed *p*-value <0.05 was considered statistically significant.

**Table 1:** Correlations between MIBS-4 and clinical/vestibular function measures (Spearman’s *ρ*).

Variable	MIBS-4 *ρ* (*p*)
Age	−0.058 (0.619)
Sex (female = 1)	0.140 (0.231)
Aura (present = 1)	−0.033 (0.779)
Headache frequency	0.353^**^ (0.002)
Non-headache days with residual symptoms	0.397^**^ (<0.001)
Clear (symptom-free) days	−0.507^**^ (<0.001)
HIT-6 score	0.414^**^ (<0.001)
Latency from migraine onset to dizziness onset	0.024 (0.839)
DHI total score	0.419^**^ (<0.001)
SDS score	0.433^**^ (<0.001)
Romberg ratio	−0.10 (0.932)
vHIT abnormal (yes = 1)	0.056 (0.633)
TUG	0.221 (0.057)
One-leg standing time (right leg)	−0.049 (0.676)
One-leg standing time (left leg)	−0.094 (0.423)
Dix–Hallpike test positive	−0.160 (0.171)

Spearman’s rank correlation coefficients (*ρ*) and *p*-values are reported.

**A *p*-value <0.05 was considered statistically significant.

HIT-6, Headache Impact Test-6; MIBS-4, Migraine Interictal Burden Scale-4; DHI, Dizziness Handicap Inventory; SDS, Situational Dizziness Score; TUG, Timed Up and Go test; vHIT, Video Head Impulse Test.

## Results

### Patient characteristics

A total of 75 patients diagnosed with VM were included in this study (Table 2). The median age was 37.0 years (IQR, 27.0–47.0), and 74.7% were female. Among them, 26.7% had migraine with aura. The median HIT-6 score was 62.0 (IQR, 58.0–65.0), and 66.7% of the patients scored ≥60, indicating a severe headache-related impact. The median MIBS-4 score was 4.0 (IQR, 1.0–7.0), with 41.3% classified as having a severe interictal burden (score ≥5). The median headache frequency was 8.0 days/month (IQR, 0.0–14.0), 6.0 unclear days/month (IQR, 3.0–10.0), and 10.0 crystal-clear days/month (IQR, 5.0–17.0). The latency from migraine onset to dizziness onset was highly variable, with a median of 120.0 months (IQR, 6.0–240.0). Regarding dizziness type, 14.7% experienced rotational vertigo, 60.0% had non-rotational dizziness, and 25.3% experienced both.

**Table 2:** Basic characteristics of the participants in this study.

Variable	Total (*n* = 75) median (IQRs)
Age, years	37.0 (27.0–47.0)
Sex, *n* (%) (male/female)	19 (25.3)/56 (74.7)
Migraine aura status, *n* (%)	
With aura	20 (26.7)
Without aura	55 (73.3)
HIT-6, points	62.0 (58.0–65.0)
HIT-6 score ≥60, *n* (%)	50 (66.7)
MIBS-4, points	4.0 (1.0–7.0)
MIBS-4 score ≥5, *n* (%)	31 (41.3)
Headache frequency, days/month	8.0 (5.0–14.0)
Non-headache days with residual symptoms, days/month	6.0 (3.0–10.0)
Clear (symptom-free) days, days/month	10.0 (5.0–17.0)
Latency from migraine onset to dizziness onset, months	120.0 (6.0–240.0)
Type of dizziness, *n* (%)	
Rotational vertigo	11 (14.7)
Non-rotational dizziness	45 (60.0)
Both	19 (25.3)

Values are presented as median (interquartile range) or number (%).

HIT-6, Headache Impact Test-6; MIBS-4, Migraine Interictal Burden Scale–4; IQR: interquartile range.

### Neurotological findings during the interictal period of migraine

Neurotological examinations conducted during the interictal period of migraine revealed a median total DHI score of 20.0 (IQR, 12.0–36.0) (Table 3), indicating a mild to moderate dizziness-related handicap. Among the VOR assessments using the v-HIT, 8.0% of patients demonstrated abnormal gain in the posterior semicircular canal. Regarding v-HIT CUS classification, 26.7% showed normal findings, while 20.0% exhibited covert saccades, 33.3% overt saccades, 13.3% corrective antisaccade quick eye movement (CAQEM), and 6.7% multiple abnormal findings. The median Romberg ratio (eye closed/eye open) was 1.45 (IQR, 1.03–1.97), and the median TUG time was 6.6 s (IQR, 5.7–7.1). One-leg standing time (eyes open) was 30.0 s bilaterally. The Dix–Hallpike test was positive in one participant (1.3%), and no positive findings were observed in the Roll test. The median SDS score was 40.0 (IQR, 35.0–48.0).

**Table 3:** Neurotological examination results.

Variable	Total (*n* = 75) Median (IQR) or *n* (%)
DHI, total score	20.0 (12.0–36.0)
Physical subscale	8.0 (4.0–12.0)
Functional subscale	4.0 (2.0–10.0)
Emotional subscale	8.0 (4.0–14.0)
vHIT abnormal gain, *n* (%)	
Lateral semicircular canal	5 (6.7)
Anterior semicircular canal	3 (4.0)
Posterior semicircular canal	6 (8.0)
vHIT CUS classification, *n* (%)	
Normal	20 (26.7)
Covert present	15 (20.0)
Overt present	25 (33.3)
CAQEM present	10 (13.3)
Multiple abnormal	5 (6.7)
Posturography	
Romberg ratio (EC/EO)	1.45 (1.03–1.97)
TUG, seconds	6.6 (5.7–7.1)
One-leg standing time (eyes open), seconds	
Right leg	30.0 (30.0–30.0)
Left leg	30.0 (30.0–30.0)
Positivity in BPPV provocation tests, *n* (%)	
Dix–Hallpike test positive	1 (1.3)
Roll test positive	0 (0)
SDS, points	40.0 (35.0–48.0)

Values are presented as median (interquartile range) or number (percentage).

vHIT catch-up saccades (CUS) were categorized as covert, overt, or CAQEM (corrective antisaccade quick eye movement).

DHI, Dizziness Handicap Inventory; SDS, Situational Dizziness Score; vHIT, Video Head Impulse Test; EC, eyes closed; EO, eyes open; TUG, Timed Up and Go test; BPPV, Benign Paroxysmal Positional Vertigo.

### Correlation and regression analyses

#### Correlation analyses

Spearman’s rank correlation analysis revealed that the MIBS-4 score was significantly positively correlated with headache frequency (*ρ* = 0.353, *p* = 0.002), unclear days (*ρ* = 0.397, *p* < 0.001), HIT-6 score (*ρ* = 0.414, *p* < 0.001), DHI total score (*ρ* = 0.419, *p* < 0.001), and SDS score (*ρ* = 0.433, *p* < 0.001) (Table 1). Conversely, the number of crystal- clear days showed a significant negative correlation with MIBS-4 (*ρ* = −0.507, *p* < 0.001). No significant correlations were found between the MIBS-4 score and age, sex, aura presence of aura, latency from migraine onset to dizziness, Romberg ratio, TUG, one-leg standing time, v-HIT, or Dix–Hallpike test results (see Table 1).

#### Multiple linear regression analyses

Stepwise multiple linear regression analysis identified the following variables as significant predictors of MIBS-4 scores: fewer crystal-clear days (*β* = −0.272, *p* = 0.015), higher HIT-6 scores (*β* = 0.265, *p* = 0.016), and higher DHI total scores (*β* = 0.250, *p* = 0.019) (Table 4).

**Table 4:** Multiple linear regression analysis of factors associated with MIBS-4 score.

Variable	Standardized *β*	95% CI	*p*-value
Clear (symptom-free) days	−0.272	−0.25 to −0.03	0.015^*^
HIT-6	0.265	0.03 to 0.27	0.016^*^
DHI total score	0.250	0.01 to 0.10	0.019^*^

Adjusted *R*^2^ = 0.335.

Standardized beta coefficients (*β*) and 95% confidence intervals (CI) were calculated using stepwise multiple linear regression analysis.

*A *p*-value <0.05 was considered statistically significant.

HIT-6, Headache Impact Test-6; MIBS-4, Migraine Interictal Burden Scale-4; DHI, Dizziness Handicap Inventory; CI, confidence interval.

## Discussion

### Patient characteristics and clinical features

In this retrospective observational study of 75 adult patients with VM, participants were predominantly female and of midlife age, with approximately one-quarter reporting migraine with aura. Most patients experienced non-rotational dizziness and moderate dizziness-related handicap. Vestibular functions were largely normal, although two-thirds of them showed abnormal CUS on the v-HIT. Only one patient tested positive on the Dix–Hallpike maneuver, and no patient had a positive Roll test. Two-thirds of patients reported a severe headache-related impact (HIT-6 score ≥60), and about 40% had a severe interictal burden (MIBS-4 ≥5), while overall depression levels were generally low. The latency from migraine onset to dizziness onset was approximately 10 years. These characteristics of the participants are consistent with previous reports (4, 5, 22). Similar to other migraine subtypes, VM exhibits a female predominance (5), with a reported female-to-male ratio between 1.5 and 5 (4). Among patients with aura, the frequency of vertigo symptoms is approximately double (22, 23). VM typically manifests later in life, often with a temporal delay following the initial onset of migraine (24). One study reported a mean interval of about 8 years between headache onset and vestibular symptoms (25). In women, vestibular symptoms may become more pronounced around the time of menopause (26). In patients with VM, these symptoms are generally triggered or exacerbated by changes in position, self-rotation, or visual motion in surrounding environment (5). They resemble those observed in a recently defined clinical entity, persistent postural-perceptual dizziness (PPPD) (27, 28).

### Vestibular functions and postural control in VM

VM is classified entirely based on patients-reported clinical features, as no biological markers currently exist (29). Although acute vestibular findings are often unavailable and interictal testing lacks specificity for diagnostic purposes, several studies have examined vestibular dysfunction in VM (6, 30–35). The v-HIT is now widely used for evaluating vestibular function and has largely replaced caloric testing in patients with suspected vestibular disorders (36). Previous studies have found that VOR gain in patients with VM during dizziness attacks does not differ from that in healthy individuals, but the number of CUS is higher in these patients, indicating peripheral vestibular involvement (30). In this study, CUS finding in v-HIT were as follows: 26.8% were normal; 20.0% exhibited covert saccades, occurring during head movement; 33.3% showed overt saccades, occurring after head movement; 13.3% showed CAQEM, characterized by quick eye movements in the direction of head movement (37); and 6.7% demonstrated multiple abnormal CUS. Previous studies have reported that patients with VM experience significant impairment of body balance during posturography testing in the interictal period (38) and exhibit greater postural instability than healthy controls (39). In the present study, although the median Romberg ratio was modestly elevated at 1.45, one out of 75 patients presented with an abnormally high Romberg ratio of 6.49, despite an MIBS-4 score of 0. This outlier likely contributed to the elevated mean Romberg ratio.

### Interictal burden and associations with dizziness-related handicap

Migraine burden, including symptoms during the headache phase (ictal burden), may persist between attacks (interictal burden) (40). HIT-6 and MIBS-4 are useful tools for gathering data and assessing ictal and interictal disability (41). Evidence suggests that patients with migraine exhibited vestibular symptoms fare worse than their counterparts (42). In our clinical practice, we assessed HIT-6 and MIBS-4 in all migraine patients at their initial visit, and observed that patients with VM experience a higher degree of interictal disability than those with migraine without dizziness. We hypothesized that subtle vestibular dysfunction in patients with VM contributes to anticipatory anxiety and reluctance to make plans to unpredictable attacks. To test this hypothesis, we used Spearman correlation and multivariable linear regression to examine the associations among MIBS-4 and DHI. MIBS-4 was positively correlated with the DHI total score, indicating that greater dizziness-related handicap accompanies high interictal burden. The Romberg ratio was not significant in univariable analysis and did not independently predict higher MIBS-4. Although impaired postural control did not significantly contribute to interictal burden in VM, these findings highlight the contributions of dizziness-related handicap to migraine interictal burden. In this study, overall depression levels assessed using the SDS were generally low, however it correlated significantly with MIBS-4, suggesting psychological factors may contribute to interictal burden.

### Therapeutic implication and management approaches

Conventional migraine preventive medications (e.g., beta-blockers, calcium channel blockers, antiepileptic drugs, acetazolamide, tricyclics and serotonin-noradrenaline reuptake inhibitors) may reduce severity and frequency of vertigo and headache in VM; however, the overall evidence for VM treatment remains limited and of low quality (43). Anti-calcitonin gene-related peptide monoclonal antibodies (e.g., erenumab, fremanezumab, and galcanezumab) have been reported to significantly reduce mean monthly dizziness and vestibular symptom days in patients with VM (44). Vestibular rehabilitation may improve vertigo symptoms, level of function, headache, and anxiety and depression symptoms in some patients with VM, particularly when symptoms are chronic incompletely resolved, or accompanied by PPPD (43).

### Limitations

This study has several limitations. First, the sample size was modest and drawn from a single headache outpatient clinic, potentially limiting statistical power and generalizability. Second, the retrospective observational design and potential residual confounding limit causal inferences between dizziness, postural instability, and interictal burden. Third, vestibular functions were assessed using limited batteries; caloric testing, vestibular-evoked myogenic potentials (VEMP), or the sensory organization test (6), were not performed, which may underscore otolith or semicircular canal dysfunction. Fourth, patient-reported measures (HIT-6, MIBS-4, DHI, SDS) are susceptible to recall and reporting biases, and test-retest reliability at the individual level was not assessed. Fifth, the timing of “interictal” testing relative to the last migraine attack was not standardized, potentially contributing to heterogeneity in physiological and symptom states. Last, although multivariable associations were explored, the use of stepwise regression with a relatively small sample size raises risks of overfitting, collinearity, and model optimism.

This study lacked a control group, limiting interpretation of the relative magnitude of interictal burden. To complement the absence of a control group, we added a description of an exploratory matched analysis using migraine patients without dizziness as control, matched for age, sex, presence or absence of aura, and monthly headache days. The supplementary analysis showed that patients with VM had significantly higher MIBS-4 score than migraineurs without dizziness (*ρ* = 0.004), despite comparable HIT-6 scores (Supplementary Table 1). These findings support the main conclusion that dizziness independently contributes to the interictal burden in VM. However, this exploratory comparison should be interpreted cautiously, and confirmation in a prospective controlled study is warranted.

## Conclusion

In patients with VM during the interictal period, vestibular functions were largely normal except for abnormalities in v-HIT CUS, whereas subjective dizziness significantly contributed to interictal disability. The dizziness observed in VM resembles that of PPPD. These findings suggest that vestibular rehabilitation may be effective in alleviating migraine interictal burden in patients with VM.

## Data Availability

The original contributions presented in the study are included in the article/Supplementary material, further inquiries can be directed to the corresponding author.
