# Supplementary material for: Impact of dizziness on migraine interictal burden in patients with vestibular migraine
Source: Front Neurol. 2025 Dec 12;16:1723725. doi: 10.3389/fneur.2025.1723725 (PMC12740907; doi:10.3389/fneur.2025.1723725)
Supplement: Supplementary file 1 [file Table_1.docx]

**Supplementary Table 1.**

| Characteristics | Control group (n=75) | VM group (n=75) | p-value | Effect size |
| --- | --- | --- | --- | --- |
| **Sex**, n (%) |  |  | 1.000 | 0.00 |
| -Male | 19 (25.3) | 19 (25.3) |  |  |
| -Female | 56 (74.7) | 56 (74.7) |  |  |
| **Age**, years | 38.0 (29.0-48.0) | 40.0 (29.0-50.0) | 1.000 | 0.01 |
| **Migraine aura status**, n (%) |  |  | 0.567 | 0.05 |
| -With aura | 16 (21.3) | 20 (26.7) |  |  |
| -Without aura | 59 (78.7) | 55 (73.3) |  |  |
| **Headache frequency**, days/month | 10.0 (6.0-15.0) | 10.0 (5.0-14.5) | 0.886 | 0.01 |
| **HIT-6**, points | 63.0 (56.5-67.0) | 62.0 (58.0-65.5) | 0.885 | 0.01 |
| **MIBS-4**, points | 2.0 (0.0-4.5) | 4.0 (1.0-8.0) | 0.004* | 0.23 |

Values are presented as median [interquartile range] or number (percentage).

Between-group differences were analyzed using the Mann-Whitney U test for continuous variables and Pearson's χ² test (or Fisher's exact test, as appropriate) for categorical variables.

A p-value < 0.05 was statistically significant (*).

Abbreviations: IQR, interquartile range; VM, Vestibular Migraine; HIT-6, Headache Impact Test-6; MIBS-4, Migraine Interictal Burden Scale–4.
